# Retro-Ileal Conduit Small Bowel Internal Herniation After Radical Cystectomy: A Surgical Complication

**DOI:** 10.7759/cureus.14142

**Published:** 2021-03-27

**Authors:** Navin Kumar, Rohik Anjum, Rishit Mani, S Chezhian, Amit Gupta

**Affiliations:** 1 General Surgery, All India Institute of Medical Sciences, Rishikesh, IND

**Keywords:** intestinal obstruction, internal herniation, radical cystectomy, ileal conduit, exploratory laparotomy

## Abstract

Intestinal obstruction is one of the most important cause of acute abdomen. An internal herniation is an uncommon yet relevant clinical entity causing an acute intestinal obstruction that can occur after major bowel surgery. Here, we describe a case of acute intestinal obstruction caused by internal herniation in a patient with muscle-invasive urinary bladder carcinoma who underwent robot-assisted radical cystectomy with an ileal conduit. We also discuss the management of adjuvant chemotherapy-induced leukopenia.

## Introduction

Intestinal obstruction is a condition that occurs when the normal flow of bowel contents is interrupted. Mechanical bowel obstruction is responsible for four out of five cases of small bowel obstruction [1]. It can be caused by extrinsic compression (tumor), intrinsic lesions (bowel neoplasms or strictures), and intraluminal obstruction (e.g., gallstone ileus, fecal impaction, foreign body). Internal hernia is a rare but important cause of mechanical bowel obstruction, requiring a high index of suspicion to diagnose. After major abdominal surgery, internal herniation is one of the differential diagnoses in a patient presenting with acute intestinal obstruction. Here, we describe a patient who presented with acute intestinal obstruction due to internal herniation in a postoperative robot-assisted radical cystectomy with an ileal conduit for urinary bladder carcinoma. We present this case in accordance with the CARE reporting checklist [2].

## Case presentation

A 39-year-old gentleman presented to the Emergency Department with complaints of abdominal distension with non-passage of flatus and feces for the past six days. He was a known case of muscle-invasive urinary bladder cancer who underwent robot-assisted radical cystectomy three months ago. The postoperative period was uneventful, and he was on adjuvant chemotherapy with gemcitabine and cisplatin and received the last cycle of chemotherapy one week before presentation to the hospital. His symptoms had gradually worsened over the period of past six days. He had average urine output from the ileal conduit. On examination, he had tachycardia (pulse rate: 110/minute) with a blood pressure of 110/70 mmHg. Abdominal examination revealed a distended abdomen with diffuse tenderness and exaggerated bowel sounds. Digital rectal examination showed collapsed rectum with no palpable mass. Clinically, he was diagnosed to have an acute intestinal obstruction.

Investigation

**Figure 1:**
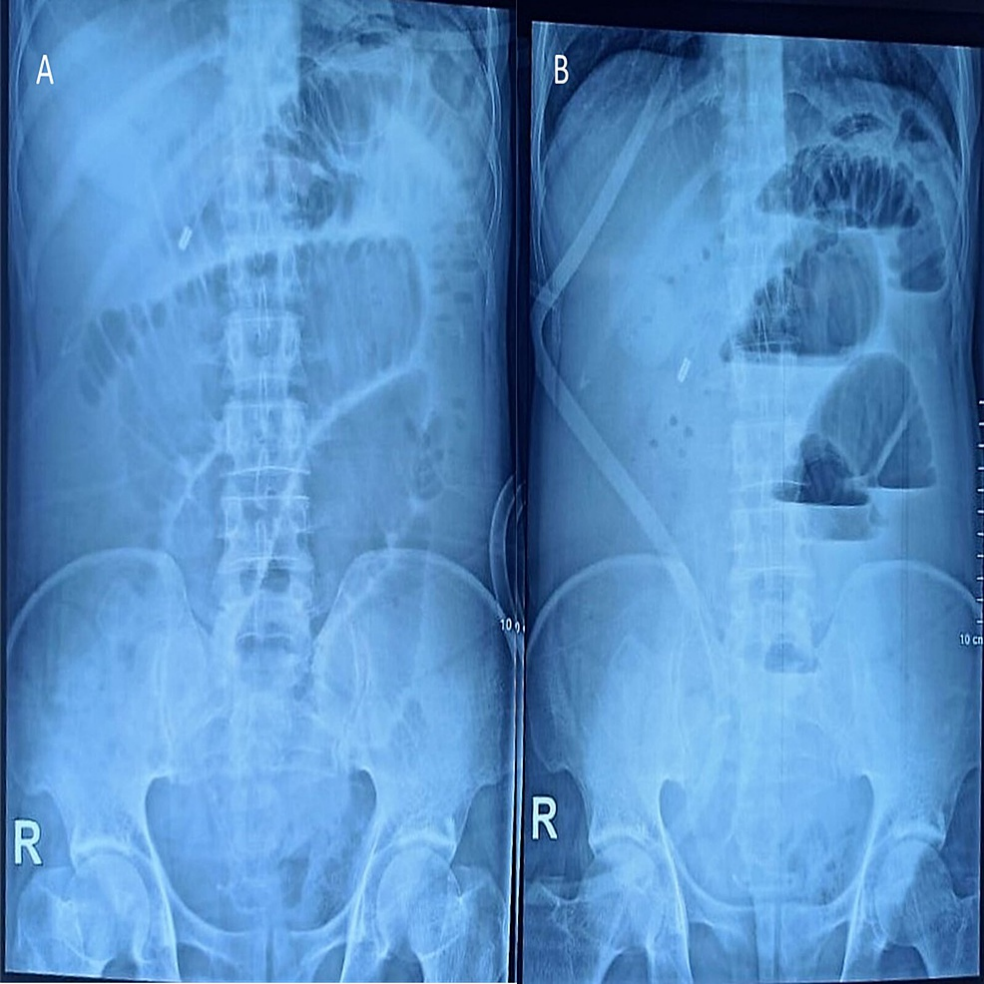
X-ray of the abdomen: supine (A) reveal dilated small bowel loops and erect (B) showing significant air fluid levels with absent gas shadow in the rectum.

**Figure 2:**
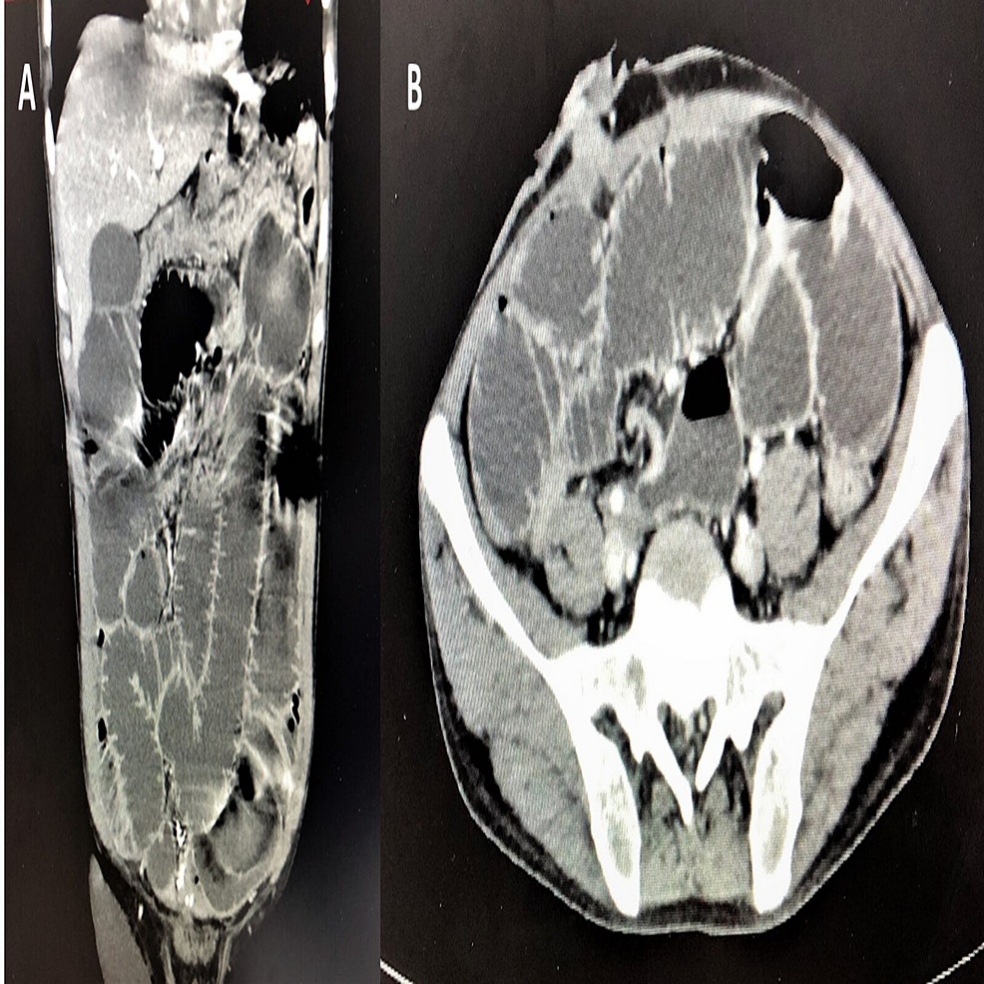
CECT of the abdomen coronal (A) and axial (B) views revealing dilated small bowel loops of jejunum and proximal ileum with transition point in the distal ileum, with collapsed large bowel. CECT: contrast-enhanced computed tomography

X-ray of the abdomen in the erect and supine position was done, which showed dilated small bowel loops with significant air-fluid levels (Figure 1). Because of suspected malignancy recurrence, he was planned for a contrast-enhanced computed tomography (CT) scan of the abdomen, which revealed dilated small bowel loops with a transition point in the mid ileum (Figure 2). Pre-operative investigations revealed leukopenia with total white blood cell (WBC) count of 1,300 cells/mm^3^ and hemoglobin of 12.2 g/dL.

Treatment

**Figure 3:**
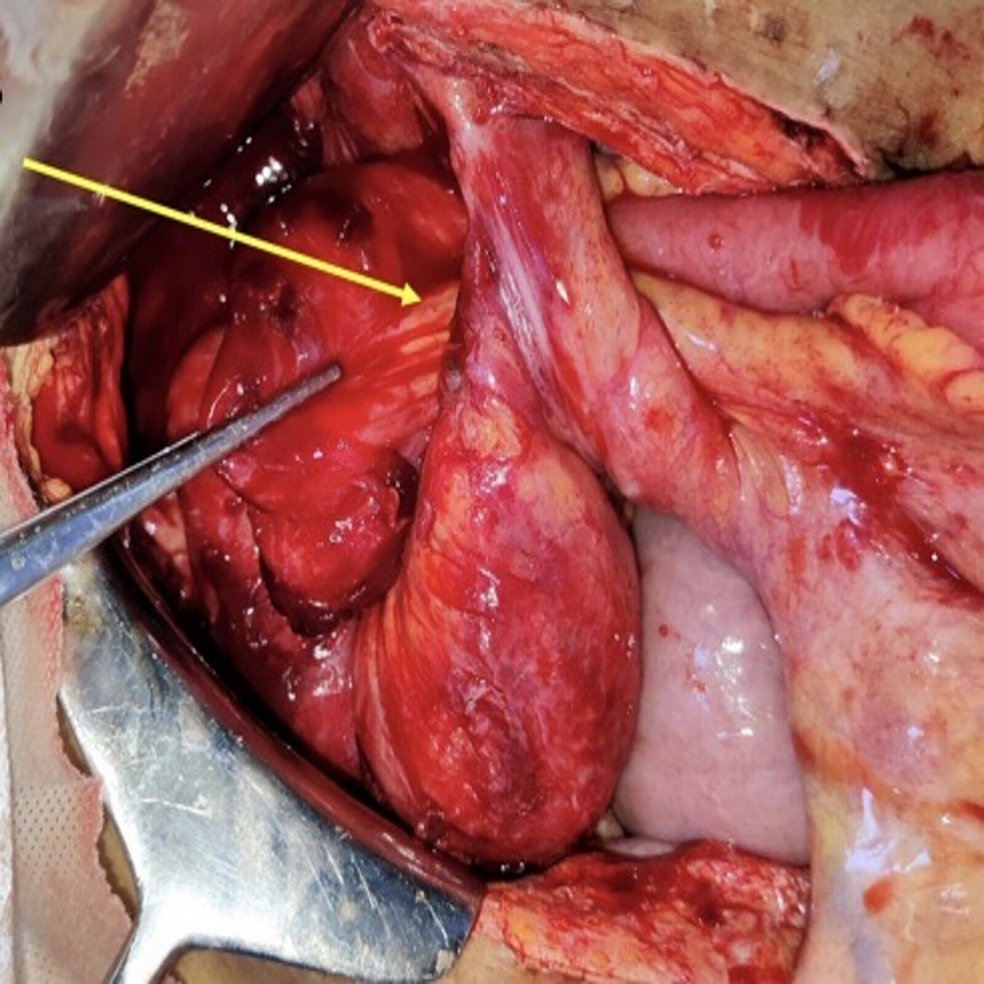
Intraoperative picture showing herniation of the ileal loop through mesenteric defect of the ileal conduit (arrow).

**Figure 4:**
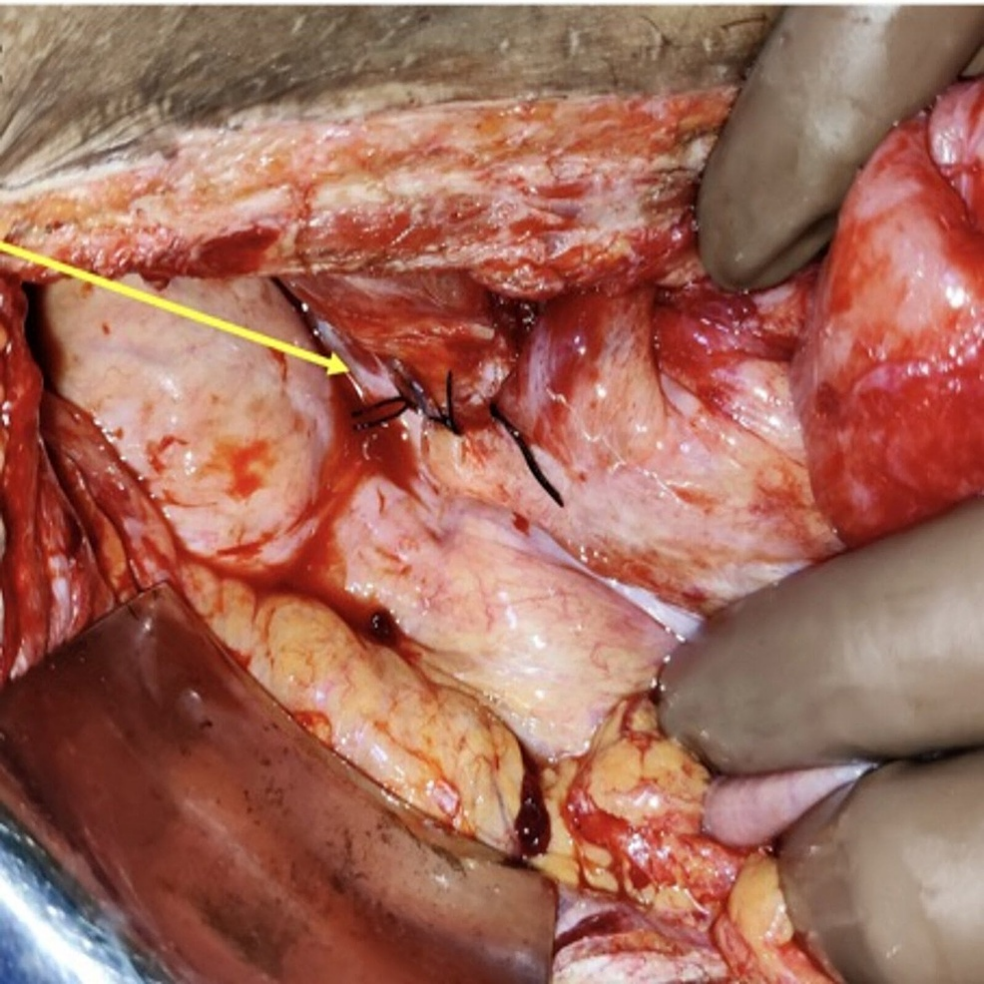
Intraoperative picture showing closure of the hernia defect (arrow).

Emergency exploratory laparotomy was performed. Dilated small bowel (jejunum and proximal ileum) was noted along with distal ileum herniating into the mesenteric defect. It had densely adhered to the defect and the right lateral pelvic wall (Figure 3). Adhesiolysis of the herniated bowel loops was done, and the bowel was reduced back to its normal anatomic position. Defect in the mesentery was closed using 2-0 silk sutures (Figure 4), and the abdomen was closed in layers. Medical Oncology opinion was taken for the chemotherapy-induced leukopenia, and the patient was administered injection granulocyte-macrophage colony-stimulating factor (GM-CSF) 300 mcg subcutaneously once daily till total count reached >4,000 cells/mm^3^.

During the postoperative period, total counts decreased further to 200 cells/mm^3^. Because of immunosuppression, broad-spectrum intravenous (IV) antibiotics (meropenem 1 g IV eight hourly and teicoplanin 400 mg IV 12 hourly followed by IV once daily), antifungal (fluconazole 100 mg IV once daily), and antiviral (acyclovir 300 mg IV once daily) were started. The patient recovered gradually, and total counts increased to 5,000 cells/mm^3^ on the fourth post-operative day (POD); he was allowed oral intake from POD three and discharged from hospital on POD seven in stable condition.

## Discussion

An internal herniation is a relatively uncommon phenomenon yet a very important cause of acute intestinal obstruction. It is the protrusion of the bowel or other viscera through a normal or abnormal orifice in the peritoneum or mesentery. It can be associated with bowel strangulation or incarceration [3]. Internal herniation is found in 0.2 to 0.9% of the cases in autopsies and cause 0.5 to 4.1% of acute intestinal obstructions [4]. Defects in internal herniation can be congenital, post-traumatic, or post-surgical. Paraduodenal hernia is the most common type accounting for 55% of the cases, followed by pericaecal (10 to 15%), foramen of Winslow (4 to 8%), and perivesicular (1 to 3%) [5].

Symptoms of patients with internal herniation are non-specific; our patient presented with a typical feature of acute intestinal obstruction because of retro-ileal conduit small bowel herniation.

The first case of internal herniation in literature causing intestinal obstruction was reported in 1997 by Kringsman et al. Their patient had a 30 cm gangrenous jejunum segment, which was resected [6]. The second published case of internal herniation was much later in 2004 by Liu et al., who performed exploration with a reduction of 20 cm of the herniated distal ileal segment without any resection [7]. A series of internal herniation following radical cystectomy was published by Tsa et al. in China, which described small bowel herniation between the two ureters. Reduction of hernia was done without any need for bowel resection, which is similar to our case [8].

Having a pre-operative diagnosis of internal herniation hastens the management of the condition. CT scan findings of internal hernias include evidence of small bowel obstruction and occurrence of closed-loop obstruction. Sac-like mass or cluster of dilated small bowel loops at an abnormal anatomic location in small bowel obstruction should alert the clinician of the possibility of internal herniation. Stretched, engorged, and displaced mesenteric vascular pedicle and convergence of vessels at the internal hernia (due to impaired venous return and continuous arterial flow) provide clues to the diagnosis [9,10]. However, this may not be identified in all circumstances, as in our case. In cases of internal herniation, prompt surgical exploration is warranted due to the high incidence of small bowel ischemia, gangrene, and perforation.

## Conclusions

Internal hernias are a rare but important cause of mechanical bowel obstruction. History of surgery or trauma are pointers toward internal herniation. Contrast-enhanced CT scan of the abdomen may be the best available emergency pre-operative investigation available for the diagnosis of internal herniation. One must ensure to close the mesenteric defect during any bowel surgeries to prevent post-operative internal herniation. In our patient, chemotherapy-induced leukopenia responded well with GM-CSF and was continued till the total WBC counts were within normal limits.
